# Case-control study on the association of angiotensin-converting enzyme gene I/D *rs*4646944 single nucleotide polymorphism and young-onset cryptogenic ischemic stroke in an Algerian cohort

**DOI:** 10.1590/1677-5449.202501422

**Published:** 2026-09-28

**Authors:** Mohamed Lebsir, Mohamed Larbi Rezgoun, Boubekeur Saddik Fekraoui, Brahim Djoudi, Yasmina Boukhenaf, Amina Ines Bernou, Karima Sifi

**Affiliations:** 1 University of Constantine 1 Mentouri – UMC1, Faculty of Life and Natural Sciences,Constantine, Algeria.; 2 University of Constantine 3, Salah Boubnider, Faculty of Medicine, Constantine, Algeria.

**Keywords:** angiotensin-converting enzyme polymorphism, cryptogenic ischemic stroke, young onset, polimorfismo ECA, acidente vascular cerebral isquêmico criptogênico, início precoce

## Abstract

**Background:**

Numerous studies have shown a link between the angiotensin-converting enzyme (ACE) and ischemic stroke. Cryptogenic ischemic stroke is the most commonly recorded form of ischemic stroke among young adults. The insertion/deletion (I/D) variant of *ACE* (*rs*4646994) has been linked to ischemic stroke.

**Objectives:**

We examined the potential correlation between this single nucleotide polymorphism and cryptogenic ischemic stroke in young adults.

**Methods:**

This case-control study included 42 young adults (< 50 years of age) diagnosed with cryptogenic ischemic stroke according to the TOAST classification, along with 42 healthy controls from the general population. The vascular risk factors included hypertension, diabetes mellitus, dyslipidemia, smoking, and cardiovascular diseases. Genomic deoxyribonucleic acid was extracted, and the *ACE rs*4646994 polymorphism was examined using polymerase chain reaction.

**Results:**

Younger age (p = 0.004) and female sex (p = 0.007) were significantly connected to an increased risk of stroke, contrasting with typical stroke demographics. Hypertension (p *=* 0.002) and smoking (p *=* 0.04) emerged as important, modifiable risk factors. There was no notable distinction between patients and controls regarding the frequencies of alleles and genotypes of the *ACE* I/D single nucleotide polymorphism (p > 0.05). These results were not associated with cryptogenic ischemic stroke.

**Conclusions:**

While aligning with the current literature, our results are constrained by the limited sample size, the case-control design, and the dependence on self-reported data, which could have led to bias. This genetic study of a population from eastern Algeria did not show a link between the *ACE*I/D single nucleotide polymorphism and cryptogenic ischemic stroke.

## Resumo

**Contexto:**

Vários estudos demonstraram associação entre enzima conversora de angiotensina (ECA) e acidente vascular cerebral (AVC) isquêmico. AVC isquêmico criptogênico é a forma mais frequentemente registrada de AVC isquêmico entre adultos jovens. A variante de inserção/deleção (I/D) da ECA (rs4646994) tem sido associada ao AVC isquêmico.

**Objetivos:**

Investigamos possível correlação entre esse polimorfismo de nucleotídeo único e AVC isquêmico criptogênico em adultos jovens.

**Métodos:**

Caso-controle incluiu 42 adultos jovens (< 50 anos) diagnosticados com AVC isquêmico criptogênico segundo a classificação TOAST, juntamente com 42 controles saudáveis da população geral. Fatores de risco vascular incluíram hipertensão, diabetes melito, dislipidemia, tabagismo e doenças cardiovasculares. Ácido desoxirribonucleico genômico foi extraído, e polimorfismo rs4646994 foi analisado por meio da reação em cadeia da polimerase.

**Resultados:**

Idade mais jovem (p = 0,004) e sexo feminino (p = 0,007) foram significativamente associados a risco aumentado de AVC, contrastando com perfil demográfico típico dessa condição. Hipertensão (p = 0,002) e tabagismo (p = 0,04) mostraram-se fatores de risco importantes e modificáveis. Não houve diferença significativa entre pacientes e controles quanto às frequências de alelos e genótipos do polimorfismo de I/D da ECA (p > 0,05). Esses resultados não demonstraram associação com AVC isquêmico criptogênico.

**Conclusão:**

Embora estejam alinhados com a literatura atual, os resultados apresentam limitações decorrentes do tamanho reduzido da amostra, do delineamento do estudo e da dependência de dados autorreferidos, fatores que podem ter introduzido viés. Este estudo, realizado em uma população do leste da Argélia, não demonstrou associação entre polimorfismo de I/D da ECA e AVC isquêmico criptogênico.

## INTRODUCTION

The high incidence, morbidity, and mortality of ischemic stroke (IS) raise concern across the world.^1,2^ Up to 25% of those affected are young, with cryptogenic IS (CIS) as the most reported subtype among this demographic.^3^ Early-onset stroke is a distinct phenotype that may help identify rare genetic variants.^4^ Risk factors can be either modifiable or non-modifiable. Non-modifiable factors include age, sex, family history, and race. In contrast, modifiable risk factors include hypertension, smoking, diet, and sedentarism.^5^ Hypertension is a crucial risk factor in IS.^6^ Epidemiological studies including families and twins have shown that stroke risk involves genetic factors.^7-9^

Vascular problems are linked to various candidate genes for stroke risk, such as angiotensin converting enzyme (*ACE*) gene.^8,10^ The *ACE* gene (OMIM: 106180) has a significant effect on vascular balance and the occurrence of atherosclerosis and hypertension. It is also thought to play a role in the onset of cerebrovascular and cardiovascular diseases.^11,12^ The *ACE* gene encodes ACE, a circulating and membrane-bound enzyme, which transforms angiotensin I into the vasoconstrictor angiotensin II and deactivates the vasodilator bradykinin, a strong vasodilator that influences various cellular functions across multiple tissues. This gene is regarded as a key candidate in cerebral small vessel disease. This is due to its critical function in managing blood pressure, vascular endothelial activity, and the growth and tension of smooth muscle.^13,14^

The *ACE* gene contains over 160 polymorphisms, primarily consisting of single nucleotide polymorphisms (SNPs).^6,15^ A case-control study of over 3,000 men reported proof of a link between the *ACE* gene and high blood pressure.^9^ In 1990, Rigat et al. reported, for the first time, 3 types of polymorphism in the *ACE* gene: homozygote deletion (DD), homozygote insertion (II), and heterozygote (ID) ^6^. Two meta-analyses that included over 100 studies found that people with D alleles had an increased susceptibility to IS.^16^ The ACE gene is located on chromosome 17q23 and has an I/D polymorphism (*rs*4646994) in intron 16.^10^ This polymorphism has been studied in relation to cerebrovascular disorders.^11,17^ Previous meta-analyses have reported a substantial link between I/D and IS risk.^12^

Until now, no study in Algeria or North Africa has attempted to investigate the potential role of the *rs*4646944 SNP in CIS. In this study, we examined the connection with this SNP in a CIS population from eastern Algeria. We assessed the distribution of genotypes and allele frequencies in CIS patients compared to healthy controls.

All procedures performed in this study followed Declaration of Helsinki guidelines. The Ethics Committee of the Dr. Benbadis University Hospital Center of Constantine approved this study (Reference Number: CE/CHUC/11/12-2024). All patients and controls provided written informed consent to participate in the study.

## METHODS

### Study Population

The study population consisted of patients from eastern Algeria, recruited over a 3-year period (2020 to 2023) from the Neurology Department of Benbadis University Hospital Center of Constantine and the local public health facility Bachir Mentouri. A total of 42 patients were recruited, all young adults (< 50 years of age), affected by CIS. The control group included 42 healthy normotensive subjects. The inclusion criteria for the study group were an IS not linked to a specific cause (e.g., atherosclerosis, cardioembolism, or small vessel disease) as per Trial of ORG 10172 in Acute Stroke Treatment (TOAST) classification. Clinical information, such as age, sex, history of heart disease, and family health records, was documented using standardized case report forms. Traditional vascular risk factors included hypertension, diabetes, dyslipidemia, smoking, and heart diseases. Hypertension was characterized by systolic blood pressure > 140 mm Hg, diastolic blood pressure > 90 mm Hg, or the use of antihypertensive drugs. Hypercholesterolemia was characterized by a plasma low-density lipoprotein cholesterol concentration ≥ 3.4 mmol/L and/or a non-high-density lipoprotein cholesterol level ≥ 4.1 mmol/L, or the use of hypercholesterolemia medication. Smoking history, including total pack years at the time of evaluation, was recorded. Diabetes was defined as either a previous diagnosis of diabetes or fasting blood glucose > 7.0 mmol/L. All participants provided written informed consent in accordance with ethical practices. The flow of participants through the study, including eligibility, exclusions, and the numbers genotyped and analyzed, is summarized in Figure 1.

**Figure 1:**
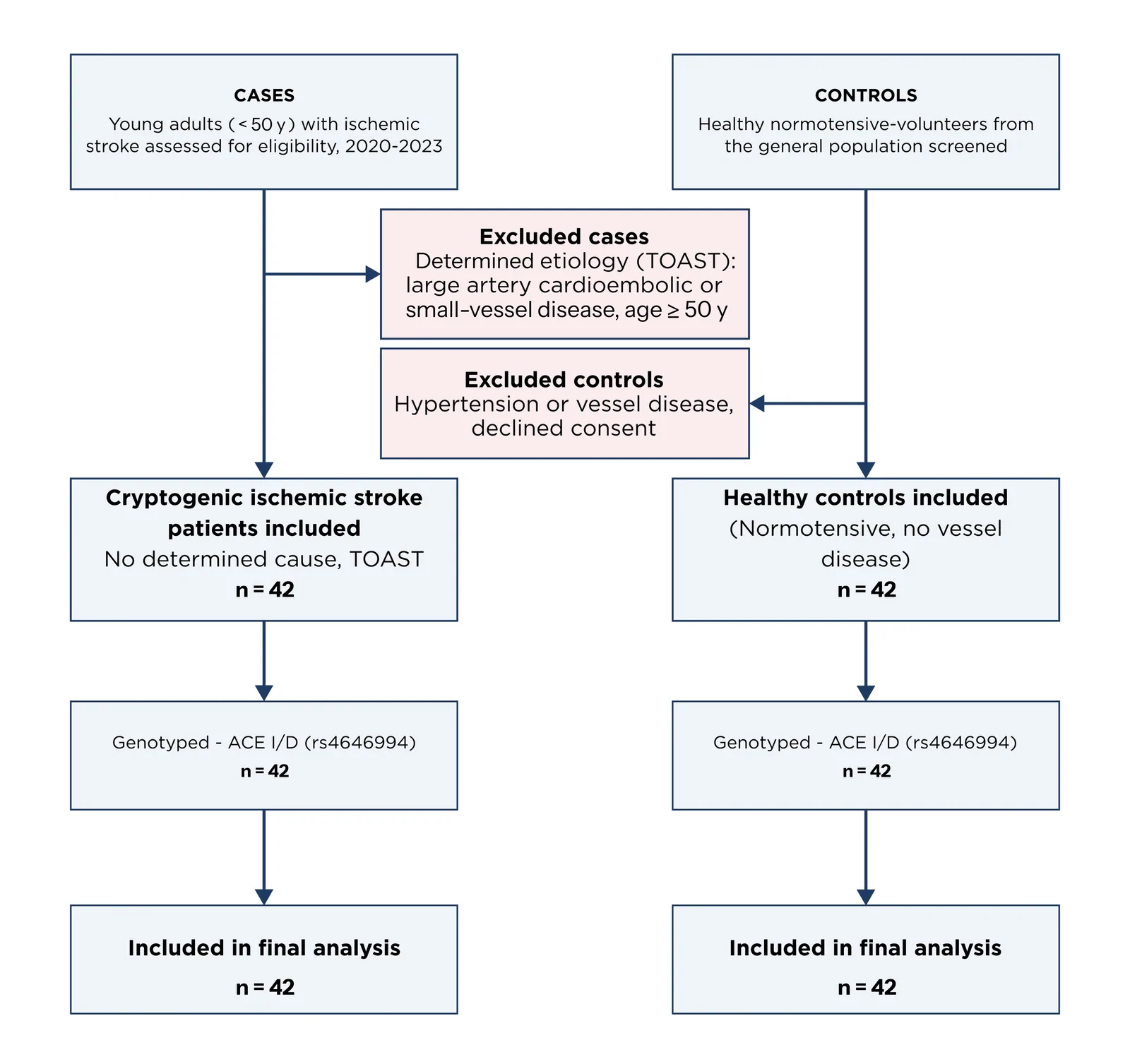
Flowchart of participant recruitment, exclusions, and final case-control population. TOAST: Trial of ORG 10172 in Acute Stroke Treatment

### Genetic analysis (angiotensin converting enzyme insertion/deletion genotyping)

#### Deoxyribonucleic acid extraction

Genomic deoxyribonucleic acid (DNA) was extracted from peripheral blood leukocytes using an inorganic solvent (salting-out technique using sodium chloride). Peripheral blood (5 ml) was collected under sterile conditions by venipuncture into a Vacutainer ethylenediaminetetraacetic acid tube. The concentration and quality of the extracted DNA were assessed using a Nanodrop 8000 (Thermo Scientific, Waltham, MA, USA).

#### Polymerase chain reaction protocol

A chain reaction amplification was performed using the following primer sequences: forward5’-CTGCAGACCACTCCCATCCTTTCT-3’ and reverse5’-GATGTGGCCATCACATTCGTCAGAT-3’in a Veriti 96-Well Fast Thermal Cycler (Applied Biosystems, Waltham, MA, USA) with the following program: primary denaturation at 95° C for 5 minutes, followed by 35 cycles of denaturation at 94° C for 30 seconds, annealing at 56° C for 40 seconds, and extension at 72° C for 30 seconds, with a final extension at 72° C for 7 minutes. The polymerase chain reaction products were observed as follows: the I allele was represented by a 490 base pair (bp) product, while the D allele was represented by a 190 bp fragment; genotype II was represented by a 490 bp fragment; the DD genotype was represented by a 190 bp fragment; and the ID genotype was represented by both a 490bp and a 190 bp fragment.

### Statistical analysis

The results were collected, organized, and analyzed using IBM SPSS Statistics 22 (IBM, Armonk, NY, USA). The Shapiro-Wilk test was used to determine the normality of distribution. All non-normally distributed parameters are presented as means (SD, medians, and interquartile ranges). The *χ*^2^ goodness-of-fit test was used to assess Hardy-Weinberg equilibrium. The frequencies of alleles and genotypes were assessed and compared between the patient and control groups with a standard *χ*^2^ test. A p value < 0.05 was deemed statistically significant. Odds ratios (OR) and 95% CI were computed to assess the influence of various alleles. Normal distribution was confirmed, and continuous variables were assessed with analysis of variance.

The minimum sample size was estimated for comparing 2 independent proportions (the frequency of the risk genotype in cases vs controls) following Miot.^18^ Considering the DD genotype frequency observed in the control group (≈78.6%) as the reference exposure, a 2-sided α of 0.05 (Zα/2 = 1.96), a statistical power of 80% (Zβ = 0.84) and a 1:1 case-to-control ratio, the detection of a moderate genetic effect (OR = 2.0, corresponding to an expected DD frequency of ≈88.0% in cases) would require approximately 242 participants per group (≈484 in total). Thus, the sample (42 cases and 42 controls) had limited statistical power (≈21%) to detect associations of this magnitude; this constraint is acknowledged among the study limitations and should be borne in mind when interpreting the lack of a significant association.

## RESULTS

### Characteristics of the study population

Our study population consisted of 42 participants (men 31%, n = 13; women 69%, n = 29). The population’s mean age was 38.31 (SD, 8.51) years, with the men being significantly older than the women (42.77 [SD, 8.11] vs 36.31 [SD, 8.03], respectively) (*p =* 0.021), suggesting a notable age difference between the sexes in the study cohort. The mean National Institutes of Health Stroke Scale score upon admission for the entire population was 5.05 (SD, 5.51), indicating mild to moderate stroke severity. There was no significant difference in National Institutes of Health Stroke Scale scores between men (4.08 [SD, 6.34]) and women (5.48 [SD, 5.16]), implying that stroke severity at admission was similar between the sexes. Comorbidity-wise, arterial hypertension was present in 21.4% of the overall cohort, with a higher occurrence in women (27.5%) than men (7.6%), although this difference was not statistically significant (*p =* 0.218). Hyperlipidemia was reported in 4.7% of the population, exclusively among women (6.9%). There was no significant difference in diabetes mellitus between men and women. Smoking was significantly more prevalent among men (46.1%) (*p =* 0.002). Migraine was reported in 47.6% of the overall patient population and 11.9% of the patients had a family history of stroke. None of our patients had dementia. (Table 1)

**Table 1:** Demographic information, baseline characteristics, and risk factors in cryptogenic ischemic stroke patients.

	All	Men	Women	*p* value
**N**	42	13 (31%)	29 (69%)	N/A
**Age (years, mean SD)**	38.31±8.51	42.77±8.11	36.31±8.03	**0.021**
**Admission NIHSS (mean SD)**	5.05±5.51	4.08±6.34	5.48±5.16	0.453
**Smoking (n, %)**	6 (14.2%)	6 (46.1%)	-	**0.002**
**Alcohol use (n, %)**	-	-	-	/
**Drug use (n, %)**	-	-	-	/
**Arterial hypertension (n, %)**	9 (21.4%)	1 (7.6%)	8 (27.5%)	0.218
**Hyperlipidemia (n, %)**	2 (4.7%)	-	2 (6.9%)	0.491
**Obesity (BMI > 30)**	5 (11.9%)	1 (7.6%)	4 (13.7%)	0.528
**Diabetes mellitus (n, %)**	2 (4.76%)	1 (7.6%)	1 (3.4%)	0.540
**Migraine**	20 (47.6%)	3 (23.08%)	17 (58.6%)	0.151
**Dementia**	-	-	-	/
**Family history of stroke**	5 (11.90%)	-	5 (17.2%)	0.181

NIHSS = National Institutes of Health Stroke Scale; BMI = body mass index; n = number. Continuous variables are expressed as mean (SD). Dashes (–) indicate no recorded cases. Values in bold are statistically significant (p < 0.05).

The patient group was significantly younger, with 71.4% being < 45 years of age, compared to only 7.1% of the control group (p < 0.05). The patient group was also predominantly female (69%) (p < 0.05). Migraine emerged as a prominent risk factor, present in 47.6% of patients but absent in controls (p < 0.05), indicating a significant connection with CIS. Overweight and a family history of stroke were also significantly more common in the patient group (11.9% for both) than the control group (p < 0.05), further implicating these factors in stroke risk. Hypertension and diabetes were more prevalent in patients (21.4% and 4.8%, respectively) than controls (7.1% and 2.4%), but not significantly so (p > 0.05). These results emphasize the distinct characteristics of cryptogenic ischemic stroke patients, characterized by younger age, female predominance, and strong associations with migraine, obesity, and family history of stroke. More research is needed to explore the fundamental mechanisms of these connections and validate them in larger groups. (Table 2).

**Table 2:** Characteristics of the cryptogenic ischemic stroke and control groups

Variables	CIS (N = 42)	Controls (N = 42)	*X^2^*	OR	CI	*p* value
**Hypertension (N, %)**						
**Yes**	9 (21.4%)	3 (7.1%)	3.500	3.545	0.886-14.184	> 0.05
**No**	33 (78.6%)	39 (92.9%)				
**Diabetes (N, %)**						
**Yes**	2 (4.8%)	1 (2.4%)	0.346	2.050	0.179-23.512	> 0.05
**No**							40 (95.2)	41 (97.6%)
**Smoking (N, %)**						
**Yes**	6 (14.3%)	7 (16.7%)	0.091	0.833	0.255-2.727	> 0.05
**No**	36 (85.7%)	35 (83.3%)				
**Migraine (N, %)**						
**Yes**	20 (47.6%)	0 (00%)	26.250	0.344	0.245-0.482	**< 0.05**
**No**						
22 (52.4%)	42 (100%)
**Obesity (N, %)**						
**Yes**	5 (11.9%)	0 (00%)	5.316	0.468	0.370-0.592	**< 0.05**
**No**	37 (88.1%)	42 (100%)				
**Family history of stroke (N, %)**						
**Yes**	5 (11.9%)	0 (00%)	5.316	0.468	0.370-0.592	**< 0.05**
**No**	37 (88.1%)	42 (100%)				

CIS = cryptogenic ischemic stroke; OR = odds ratio. Values in bold are statistically significant (p < 0.05).

### Genotyping results

This investigation of *ACE* gene polymorphism detected 2 *ACE* genotypes: the DD homozygote and the ID heterozygote. The DD genotype was the most prevalent, followed by the ID heterozygote; no cases of the II homozygote were observed. (Figure 2).

**Figure 2:**
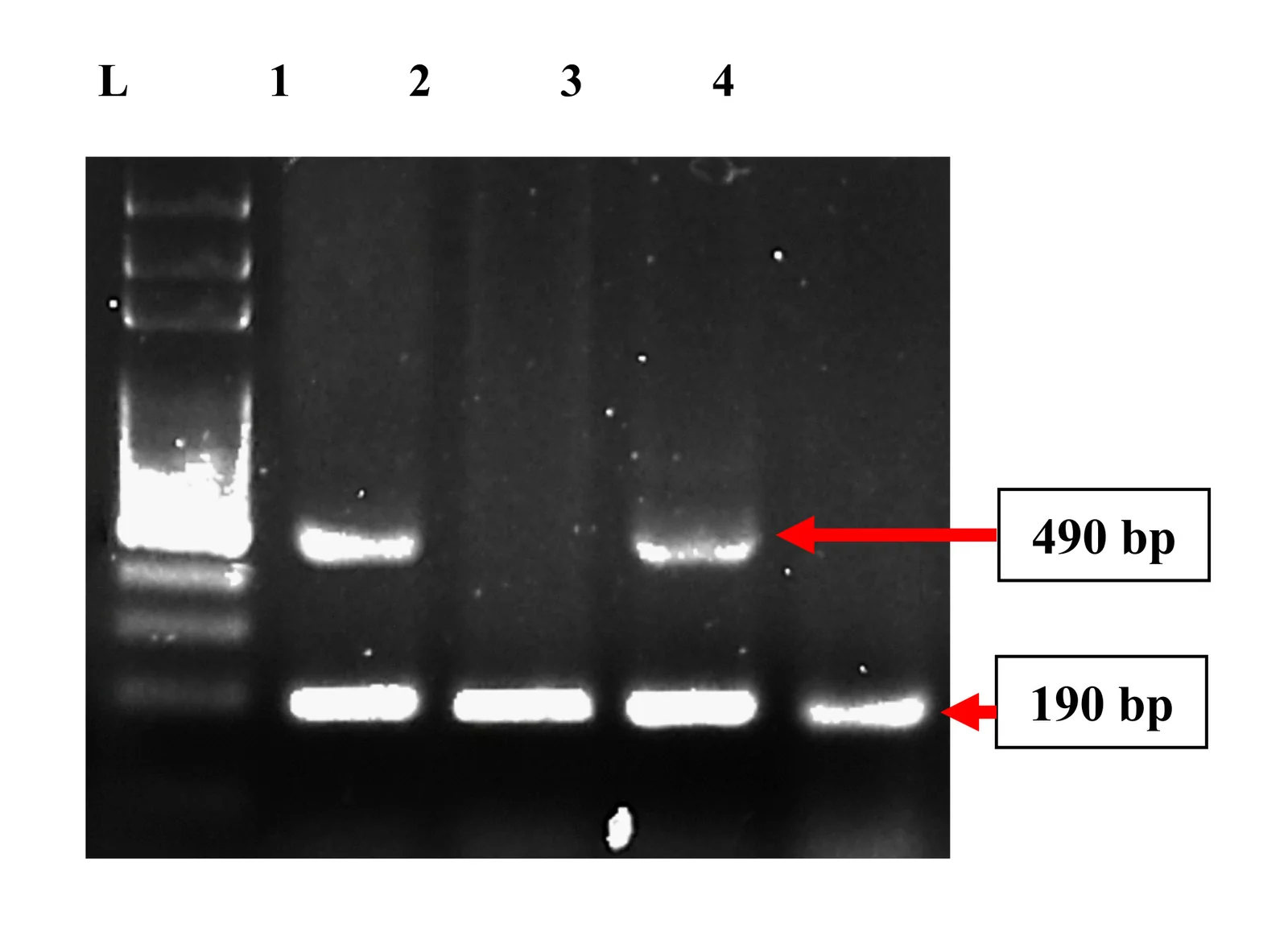
Agarose gel electrophoresis of the *ACE* I/D polymorphism PCR product. Lane L: Ladder (100 bp) – lanes 1 and 3 are heterozygous for the I and the D alleles and lanes 2 and 4 are homozygous for the D allele.

The Hardy-Weinberg equation results indicate that the genotypes of the studied SNPs were in Hardy-Weinberg equilibrium in both the patient (p *=* 0.4951) and control (p *=* 0.4367) groups (*p*> 0.05). Genotypic and allelic distribution are described in Table 3.

**Table 3:** Genotype and allelic distributions of the angiotensin converting enzyme single nucleotide polymorphism in the cryptogenic ischemic stroke and control groups

		**CIS** **N (%)**	**Control** **N (%)**	***X^2^*(df)**	***p-*value**	**OR (95%CI)**
Genotypes	DD	34 (80.95)	33 (78.57)	0.074(1)	0.786	1.159 (0.399-3.366)
	ID	08 (19.05)	09 (21.43)	0.074(1)	0.786	0.863 (0.297-2.505)
	II	-	-	-	-	-
Allele	D	76 (90.48)	75 (89.29)	0.065(1)	0.798	0.877 (0.321-2.395)
	I	08 (09.52)	09 (10.71)			

Df = degrees of freedom; OR = odds ratio. The II genotype was not observed in either group and both groups were in Hardy–Weinberg equilibrium (p > 0.05).

DD was the most predominant genotype in both the patient (81%) and the control (78.6%) groups, followed by the ID genotype (19% in patients and 21.4% in controls). The II genotype was absent in both groups. There was no significant variation in genotype distribution between patients and controls (*p*> 0.05). The OR for the DD genotype was 0.786, suggesting no notable connection between the DD genotype and CIS (Table 3).

## DISCUSSION

This study primarily investigated the link between the *rs*4646944 polymorphism of the *ACE* gene and a genetic tendency toward CIS in a group of Algerian patients. The *ACE* gene has been suggested as an independent genetic contributor to hypertension and various cardiovascular diseases.^19^ ACE converts angiotensin I into angiotensin II; elevated levels and activity of angiotensin II activate the angiotensin II type 1 receptor, subsequently inducing nicotinamide adenine dinucleotide phosphate oxidase activation, a major source of reactive oxygen species. The resulting oxidative stress reduces nitric oxide bioavailability, leading to endothelial dysfunction and impaired vasodilation. Furthermore, increased angiotensin II signaling promotes a pro-inflammatory and prothrombotic state through the upregulation of plasminogen activator inhibitor-1 and endothelial adhesion molecules, thereby facilitating thrombotic activity and cerebral microvascular injury. Therefore, ACE is regarded as a significant element in cerebrovascular disease risk.^20^ Hypertension is recognized as the primary risk factor for IS, and a number of studies have indicated a correlation between the *ACE* I/D SNP and both ischemic and hemorrhagic stroke. Nevertheless, these outcomes might differ based on the racial composition of the research population.^21,22^

As reported in previous studies, we found that hypertension, a modifiable risk factor, is significantly correlated with IS risk (p *=* 0.002), (OR = 0.375).^12^ We also found strong associations between CIS and migraine (p < 0.05), (OR = 0.344); such a finding is similar to the results of a study on young patients with CIS.^16^ Our findings showed that younger age was significantly associated with CIS (p < 0.05), as 30 patients were under 45 years old, corroborating the results (mean age 41.1 [SD, 7.9] years) of another study.^3^ Moreover, the patient population was mainly female. Several studies involving participants from diverse racial backgrounds have demonstrated a significant association between smoking and early-onset IS, and we confirmed this finding, as tobacco consumption was found to be a significant risk factor (p *=* 0.04)^23^ (Table 4).

**Table 4:** Statistical analysis of cryptogenic ischemic stroke risk factors.

Risk factor	*p* value	OR	95% CI	
			Lower	Upper
**Age**	**0.004**	**0.421**	**0.233**	**0.760**
**HTN**	**0.002**	**0.375**	**0.203**	**0.693**
**Migraine**	0.08	0.42	0.230	2.150
**Smoking**	**0.04**	**4.364**	**1.017**	**18.730**
**Sex**	**0.007**	**0.347**	**0.160**	**0.750**
***ACE* Mutation**	0.766	0.762	0.127	4.567
**Diabetes mellitus**	0.907	1.190	0.063	22.468
**Obesity**	0.07	1.079	0.000	7.329
**Family history of stroke**	0.09	0.31	0.000	0.999

OR = odds ratio; HTN = arterial hypertension; ACE = angiotensin-converting enzyme. Values in bold indicate statistical significance (p < 0.05).

The primary pathophysiology of hypertension involves the renin-angiotensin aldosterone system, with angiotensin-converting enzyme playing a central role. Consequently, the *ACE* gene is a logical candidate for genetic studies, further reinforcing its selection as a candidate gene for investigation.^7^ The *ACE* I/D gene variation influences ACE activity. The serum concentration and activity of ACE are higher in individuals with the DD polymorphism than in those with the II polymorphism, while ID heterozygotes are at an intermediate level.^6^ We found the DD genotype to be more present in the patient population, which is consistent with literature,^24,25^ although there were no significant differences between groups. The lack of association between the *ACE* I/D polymorphism and hypertension suggests that conflicting results across populations may stem from complex gene–environment interactions.^26^

To the best of our knowledge, no research has specifically assessed the effects of the I/D SNP in the *ACE* gene in individuals with CIS. Nevertheless, certain findings are somewhat akin to ours, indicating that the incidence of stroke is not meaningfully associated with *ACE* polymorphisms.^27^ In a Mexican sample, *ACE* variation showed an ID genotype frequency of 47.3% in IS patients vs 46% in controls. A greater percentage of the II genotype was observed (35.3%) in comparison to our findings, where genotype II was completely absent in CIS patients and controls.^28^ Stankovic et al.^29^ reported that the frequency of *ACE* genotypes or alleles did not significantly differ between IS patients and healthy individuals, although those with the D allele had a higher risk of large vessel IS. Murali et al. demonstrated a notable link between the II polymorphism and the I allele in men aged > 50 years with IS in a sample from southern India^6^. However, Vijayan et al.^30^ found that ID *ACE* polymorphisms are associated with stroke onset in women. Yadav et al.^31^ reported a significantly higher risk associated with the *ACE*/DD variant among South Asians (OR = 5.00; 95% CI: 1.17–21.37), indicating a 5-fold increase in the likelihood of IS per copy of the risk allele, compared to a modest risk observed in Europeans (OR = 1.15; 95% CI: 1.06–1.25). According to Kalita et al.^32^, the DD genotype poses a higher risk for Asian patients suffering from small vessel disease. Moreover, Das et al.^33^ demonstrated that the DD genotype and D allele increase the risk of stroke, particularly hemorrhagic stroke. Conversely, in Turkish populations, no relationship have been found between IS or subtypes and the DD genotype and/or D allele of the *ACE* polymorphism.^34^ Comparable outcomes were reported by Seckin et al.^35^ in a Spanish population and by Karagiannis et al.^36^ in a Greek population. Mostafa et al.^6^ examined *ACE* polymorphism in Egyptian patients with IS, subsequently indicating that the DD genotype was more prevalent in the patient group, without specificity to large or small vessel disease. In a Polish population, Pera et al.^37^ found no connection between the etiologic form of IS and *ACE* I/D polymorphism.^38^

The frequency of *ACE* polymorphisms varies across populations, with the D allele being more prevalent in Caucasians, while the I allele is further prevalent among South American and Polynesian populations.^39,40^ Among ordinary Algerians, all previous case-control studies have indicated that in *ACE* I/D SNP and control populations, the frequency of the I allele is very low and that the DD genotype is always more predominant than the ID and II genotypes.^41-43^

A methodological limitation of conventional polymerase chain reaction-based genotyping of the *ACE* I/D polymorphism should be considered when interpreting our findings. Due to preferential amplification of the smaller D allele fragment (190 bp) over the larger I allele fragment (490 bp), ID heterozygotes may occasionally be misclassified as DD homozygotes. Consequently, the complete absence of the II genotype observed in our cohort should be interpreted cautiously, as this technical bias may have contributed to an underestimation of the I allele frequency in this population.^44^

Genetic influence may lead to the occurrence of stroke through predisposition, modifiable risk factors, or both, by directly affecting the risk, progression, and outcomes associated with stroke. Racial variations among populations could result from different distribution frequencies of ACE polymorphisms, stroke types, specific matching criteria, and selection bias.^45,46^

The present study provides important insights into the clinical and biological characteristics of early-onset CIS in Algeria, highlighting the impact of conventional vascular risk factors and possible genetic influences. It also highlights the importance of comprehensive risk factor management and the integration of genetic information. Further research is required to elucidate the molecular mechanisms linked to CIS and discover new therapeutic targets likely to prevent the occurrence of CIS, or at least improve the results of therapeutic monitoring for these patients.

## CONCLUSIONS

Our findings reveal several key insights into the risk factors associated with CIS. Younger age (p *=* 0.004) and female sex (p *=* 0.007) were strongly related to a higher risk of stroke, contrasting with typical stroke demographics. Hypertension (p *=* 0.002) and smoking (p *=* 0.04) emerged as strong, modifiable risk factors, with smokers being > 4 times more likely to develop CIS than non-smokers. Migraine, obesity, and family history of stroke showed trends toward significance, suggesting potential roles in stroke risk that warrant further investigation. In contrast, *ACE* gene polymorphism (*rs*4646994) and diabetes mellitus were not significantly related to stroke risk, indicating they are unlikely to contribute to CIS in this population. Consequently, in this study, we examined a single SNP, which rules out neither the participation of other genetic variants in the *ACE* gene nor dependence on self-reported data, which could also lead to a certain degree of bias. A larger replication study with additional polymorphic variants of the gene could provide clearer insights into the function of this gene in CIS.

These results show the importance of dealing with modifiable risk factors, like high blood pressure and smoking. They also show the importance of further research into the specific demographic and clinical profiles of CIS patients, especially the role of migraines, obesity, and family history of stroke. Finding shared gene variations that could increase the likelihood of CIS is crucial for public health, as it may assist in identifying and treating subpopulations at higher risk of the disease.

## Data Availability

All data generated or analyzed are included in this article and/or in the supplemental material.

## LIST OF ABBREVIATIONS

ACE: Angiotensin Converting Enzyme

CIS: Cryptogenic Ischemic Stroke

DNA: Deoxyribonucleic acid

I/D: Insertion/Deletion

IS: Ischemic Stroke

OR: Odds ratio

SNP: Single Nucleotide Polymorphism
